# A Cross-Sectional Study on the Prevalence of Self-Prescribed Aspirin Use for Prevention of Adverse Ischemic Vascular Events Among Moderate-Risk Individuals: A Single Center Study

**DOI:** 10.7759/cureus.33531

**Published:** 2023-01-09

**Authors:** Nasser A Almulhim, Fahad K Al Mulhim, Ali H Al Nasser, Khurayzan F Bin Sifran, Mohammed A AlDabbab, Mohammad N Almulhim, Malak I Sabeela, Fatimah A Bomouzah, Omar A Aldamigh, Mohammed H Alghamdi

**Affiliations:** 1 Emergency Medicine, King Fahad Hospital, Al-Hofuf, SAU; 2 Emergency Medicine, King Fahad Specialist Hospital - Dammam, Dammam, SAU; 3 Internal Medicine, King Fahad Hospital, Al-Hofuf, SAU; 4 College of Medicine, King Faisal University, Al-Hofuf, SAU

**Keywords:** emergency room, secondary prevention, primary prevention, cardiovascular disease, aspirin

## Abstract

Introduction

Despite the overwhelming amount of evidence against the use of aspirin for primary prophylaxis of cardiovascular disease (CVD), the arguably unwarranted use of aspirin has increased over the years, which may or may not be based on any specific medical advice, and merely self-prescribed.

Aim

This study aimed to determine the prevalence of unwarranted aspirin use among moderate-risk individuals for the primary prevention of adverse vascular events in Saudi Arabia.

Patient and method

A cross-sectional study was conducted among 100 patients who presented to the emergency room (ER) due to suspected cardiac diseases. The data were collected from the patients who visited ER in King Fahad Hospital, Al-Hofuf, Saudi Arabia. Participants were asked about their socio-demographic characteristics, aspirin use habits, and their comorbidities.

Results

Of the 100 patients, 35% were aged more than 60 years old. The overall use of aspirin was 78%. The prevalence of aspirin use was significantly more common among the older age group (53.8%), those with associated chronic diseases, such as diabetes (59%) and hypertension (75.6%) and those with a previous history of hospitalization due to stroke or a cardiac event (66.7%).

Conclusion

The use of aspirin among patients who are at moderate risk of cardiovascular events was widely prevalent, but their unwarranted use was low. Older patients with chronic diseases who believed in its great benefit and tended to ignore its complications are the most common users of aspirin. More research is recommended to determine the prevalence and the factors associated with unwarranted use of aspirin in patients with CVD in our region.

## Introduction

Cardiovascular disease (CVD) is the leading cause of death in the United States, especially among patients with co-morbidities like chronic kidney disease and diabetes mellitus [1]. The prevention of CVD should be tittered to patients individually, but it generally involves controlling risk factors, lifestyle modifications, and various medications [2]. The medication of interest in this article is aspirin, an anti-platelet aggregation agent used in the prevention of ischemic heart disease and stroke [3,4].

Aspirin’s mechanism of action involves several different pathways depending on its dosage. Lower doses of aspirin cause suppression of COX-1 which, in turn, results in the suppression of platelet biosynthesis of TXA2, which plays a crucial role in platelet aggregation and primary hemostasis [3]. Higher doses of aspirin result in COX-2 inhibition in addition to COX-1, which leads to reduced production of prostacyclin and prostaglandin E, which gives aspirin its analgesic and antipyretic properties, but that can also result in vasoconstriction, renal dysfunction, and hyponatremia [5].

The role of aspirin in secondary prophylaxis CVD is well established in high-risk patients, as the benefits of low-dose aspirin clearly outweigh the risk of complications [3,6]. While an argument can be made for the use of low-dose aspirin in primary prophylaxis in high-risk individuals [7], its use in primary prevention of CVD in low-risk individuals is very controversial, most studies suggest that the use of aspirin in primary prevention yields a non-significant to a very modest absolute reduction in adverse ischemic vascular events along with a slightly increased risk of hemorrhagic events [3,6,8-10].

Despite the overwhelming amount of evidence against the use of aspirin for primary prophylaxis of CVD, the arguably unwarranted use of aspirin has increased over the years [4], which may or may not be based on any specific medical advice, and merely self-prescribed.

## Materials and methods

This was a cross-sectional study conducted in a period from September 2021 to May 2022 and aimed to determine the prevalence of unwarranted aspirin use among moderate-risk individuals for primary prevention of adverse vascular events in Saudi Arabia. Ethical approval was granted from the Institutional Review Board committee of King Fahad Hospital in Al-Hofuf, and consent was obtained from all participants prior to data collection.

The study was held in the emergency department of King Fahad Hospital in Al-Hofuf. Convenience sampling was used as the sampling technique, data were obtained from consenting patients who presented to the emergency department with chest pain and were suspected of having cardiac disease.

Participants of the study were interviewed and were asked a sequence of questions and a questionnaire was filled with their answers. The questionnaire started with a set of questions about the participants’ demographical data including age, sex, and if the participants had any chronic disease. The next section of the questionnaire was regarding whether participants were regularly taking aspirin or not, and if so, it was followed with a series of questions regarding the dose they take, the timing, relation to meals, whether aspirin was taken as per a physician recommendation or not, along with the participants’ knowledge regarding the usefulness and complications of aspirin use. The next part of the questionnaire explored participants’ attitudes towards aspirin use with questions exploring whether participants believed the benefits of taking aspirin in their case outweighed the risks and whether they were aware of some of the adverse effects that may be caused by aspirin such as peptic ulcer disease. The remainder of the questionnaire contained questions that explored whether participants had been admitted for acute chest syndrome or strokes. The questionnaire concludes with data regarding some of the tests done in the hospital to determine if participants were suspected to have had acute coronary syndrome at the time of presentation.

The data were analyzed using Statistical Packages for Social Sciences (SPSS) version 26 (IBM Corp., Armonk, NY, USA). Descriptive statistics were summarized using numbers and percentages. The use or plan to use Aspirin was compared with the socio-demographic characteristics and the awareness of potential complications of unwarranted use of Aspirin by using the Fischer Exact test. A p-value cut-off point of 0.05 at 95% CI was used to determine statistical significance.

## Results

A total of 100 patients were able to complete the survey. As seen in Table 1, 35% were older than 60 years of age with 65% of the participants being female. Most patients were married (90%) and 20% were illiterate. Approximately, 46% reported having a sedentary lifestyle, and only 21% reported achieving good control over their comorbidities. Approximately, 17% were smokers. The proportion of patients who have had a family history of heart-related deaths was 22%.

**Table 1:** Socio-demographic characteristics of the patients (n=100)

Study data	N (%)
Age group	
24 – 40 years	17 (17.0%)
41 – 50 years	18 (18.0%)
51 – 60 years	30 (30.0%)
>60 years	35 (35.0%)
Gender	
Male	65 (65.0%)
Female	35 (35.0%)
Marital status	
Single	05 (05.0%)
Married	90 (90.0%)
Divorced or widowed	05 (05.0%)
Educational level	
Uneducated	20 (20.0%)
Primary school	16 (16.0%)
Middle school	17 (17.0%)
High school	21 (21.0%)
Diploma	02 (02.0%)
University degree	14 (14.0%)
Postgraduate	10 (10.0%)
How would you describe your level of activity?	
Active (5-7 times per week)	26 (26.0%)
Somewhat active (4-2 times per week)	26 (26.0%)
Sedentary (<2 times per week)	46 (46.0%)
Others	02 (02.0%)
How well are your co-morbidities managed?	
Completely out of control	04 (04.0%)
Somewhat out of control	13 (13.0%)
Neutral	38 (38.0%)
Somewhat under control	24 (24.0%)
Completely under control	21 (21.0%)
Do you smoke?	
Yes, I currently smoke or smoked within the last 6 months.	17 (17.0%)
No, I have never smoked.	62 (62.0%)
No, I quit smoking more than 6 months ago.	21 (21.0%)
Family history of heart-related deaths	
Yes	22 (22.0%)
No	67 (67.0%)
Not sure	11 (11.0%)

In Table 2, the prevalence of patients who were taking aspirin regularly was 65%. Among those who regularly take aspirin, 94.9% were taking aspirin upon physician recommendation and 75.6% were taking aspirin during or after meals.

**Table 2:** Characteristics of patients regarding the use of Aspirin (n=100)

Variables	N (%)
Taking Aspirin regularly	
Yes, I currently take aspirin.	65 (65.0%)
No, but I plan to do so in the future.	02 (02.0%)
I used to take aspirin but stopped on my own terms.	06 (06.0%)
I used to take aspirin but stopped based on a doctor's recommendation.	05 (05.0%)
No, and I don't plan to take it in the future.	22 (22.0%)
If you take aspirin or plan on taking aspirin, who recommended it to you? (n=78)	
Physician	74 (94.9%)
Non-physician	04 (05.1%)
If you take aspirin, what dose of aspirin do you take? (n=78)	
81 mg	30 (38.5%)
100 mg	16 (20.5%)
500 mg	01 (01.3%)
I don't know	31 (39.7%)
If you take aspirin, how many pills are the doses divided over? (n=78)	
Half a pill (per day)	04 (05.1%)
One (per day)	67 (85.9%)
Two (per day)	03 (03.8%)
Others	04 (05.1%)
If you take aspirin, when do you take the pills? (n=78)	
Morning (6 am - 11 am)	37 (47.4%)
Noon/Afternoon (11 am - 4 pm)	17 (21.8%)
Evening (4 pm - 7 pm)	03 (03.8%)
Night (after 7 pm)	13 (16.7%)
Others	08 (10.3%)
If you take aspirin, do you take the pills before or after meals? (n=78)	
Before meals	11 (14.1%)
During or after meals	59 (75.6%)
Unrelated to meals	04 (05.1%)
Others	04 (05.1%)

In Table 3, more than half of the participants (54%) presented to the ER with chest pain that was highly suspected of being of cardiac origin. Only 8% showed a significant ST-elevation and only 2% had more than three times normal troponin levels.

**Table 3:** Characteristics of the patients during ER presentation

Statement	N (%)
Did the patient present with chest pain?	
Yes, highly suspicious for cardiac origins	54 (54.0%)
Yes, moderately suspicious for cardiac origins	34 (34.0%)
Yes, but only slightly/unlikely to be cardiac in origin	09 (09.0%)
No, the patient did not present with chest pain	03 (03.0%)
History of any of the following ECG changes?	
Significant ST-Elevation	08 (08.0%)
Significant ST-Depression	02 (02.0%)
Non-specific changes	57 (57.0%)
No history of ECG changes	29 (29.0%)
Others	04 (04.0%)
Troponin levels	
> 3x normal limit	02 (02.0%)
1 - 3x normal limit	08 (08.0%)
Within normal limit	62 (62.0%)
Not measured	20 (20.0%)
Pending	01 (01.0%)
Others	07 (07.0%)

**Figure 1:**
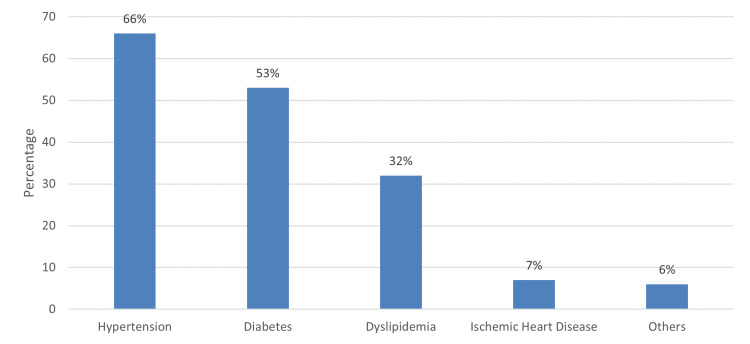
Associated chronic disease

Figure 1 shows the associated chronic disease that ailed the participants. It can be observed that the most commonly seen chronic disease among participants was hypertension (66%), followed by diabetes (53%) and dyslipidemia (32%).

Table 4 shows that 43% of participants believed that the benefits associated with taking aspirin outweigh the potential risks, and 21% of participants were aware that peptic ulcers are a possible complication of this medication. Only 7% were aware that some drugs are not recommended to be taken with aspirin. The percentage of participants who had been hospitalized due to stroke or a cardiac condition was 58% with the most common cause being coronary artery disease (51.7%). The proportion of participants who had a close relative diagnosed with a stroke or a cardiac condition was 48%, with heart disease being the most commonly seen disease among them (37.5%).

**Table 4:** Awareness of the potential complication of the use of Aspirin

Statement	N (%)
Believed the benefits associated with taking aspirin outweigh the potential risks	
Yes	43 (43.0%)
No	19 (19.0%)
Not sure	38 (38.0%)
Are you aware that peptic ulcers are possible complications of this medication?	
Yes	21 (21.0%)
No	40 (40.0%)
Maybe	39 (39.0%)
Do you know some drugs are not recommended to be taken with aspirin?	
Yes	07 (07.0%)
No	51 (51.0%)
Maybe	42 (42.0%)
Have you been hospitalized for a stroke or a cardiac condition before?	
Yes	58 (58.0%)
No	34 (34.0%)
Not sure	08 (08.0%)
If the answer to the previous question is yes, please specify (n=58)	
Coronary Artery Disease	30 (51.7%)
Stroke	07 (12.1%)
Open heart surgery	04 (06.9%)
Valvular Heart Disease	03 (05.2%)
Others	14 (24.1%)
Has a close relative been diagnosed with a stroke or a cardiac condition before?	
Yes	48 (48.0%)
No	40 (40.0%)
Not sure	12 (12.0%)
If the answer to the previous question is yes, please specify the disease (n=48)	
Coronary Artery Disease	15 (31.3%)
Stroke	15 (31.3%)
Heart disease	18 (37.5%)

When measuring the relationship between the use of aspirin (including those planning to use it) in regard to the socio-demographic characteristics of the patients as shown in Table 5, it was found that the prevalence of patients who used or were planning to use aspirin was significantly more common among the older age group (53.8%), those with associated chronic diseases, such as diabetes (59%) and hypertension (75.6%) and those with a previous history of hospitalization due to stroke or a cardiac event (66.7%).

**Table 5:** Relationship between the use or planning to use Aspirin according to the socio-demographic characteristics and the awareness of the potential complication * Some patients have multiple chronic diseases. § P-value has been calculated using Fischer Exact test. ** Significant at p<0.05 level.

Factor	Use or plan to use Aspirin		P-value ^§^
	Yes, N (%) (n=78)	No, N (%) (n=22)	
Age group			
≤55 years	36 (46.2%)	18 (81.8%)	0.003 **
>55 years	42 (53.8%)	04 (18.2%)	
Gender			
Male	50 (64.1%)	15 (68.2%)	0.804
Female	28 (35.9%)	07 (31.8%)	
Educational level			
High school or below	60 (76.9%)	14 (63.6%)	0.210
Diploma or higher	18 (23.1%)	08 (36.4%)	
Level of physical activity			
Active	44 (56.4%)	08 (36.4%)	0.146
Inactive	34 (43.6%)	14 (63.6%)	
Associated chronic disease *			
Diabetes	46 (59.0%)	07 (31.8%)	0.030 **
Hypertension	59 (75.6%)	07 (31.8%)	<0.001 **
Dyslipidemia	28 (35.9%)	04 (18.2%)	0.130
Ischemic heart disease	07 (09.0%)	0	0.342
Others	04 (05.1%)	02 (09.1%)	0.610
How well are your co-morbidities managed?			
Uncontrolled	12 (15.4%)	05 (22.7%)	0.012 **
Neutral	25 (32.1%)	13 (59.1%)	
Controlled	41 (52.6%)	04 (18.2%)	
Do you smoke?			
Yes	12 (15.4%)	05 (22.7%)	0.520
No	66 (84.6%)	17 (77.3%)	
Believed the benefit of taking aspirin			
Yes	43 (55.1%)	0	<0.001 **
No	18 (23.1%)	01 (04.5%)	
Not sure	17 (21.8%)	21 (95.5%)	
Awareness that peptic ulcers are possible complications			
Yes	21 (26.9%)	0	<0.001 **
No	37 (47.4%)	03 (13.6%)	
Maybe	20 (25.6%)	19 (86.4%)	
Knowledge of some drugs that are not recommended to be taken with aspirin			
Yes	07 (09.0%)	0	<0.001 **
No	50 (64.1%)	01 (04.5%)	
Maybe	21 (26.9%)	21 (95.5%)	
Been hospitalized due to stroke or cardiac event			
Yes	52 (66.7%)	06 (27.3%)	0.003 **
No	21 (26.9%)	13 (59.1%)	
Not sure	05 (06.4%)	03 (13.6%)	

## Discussion

This study investigated the prevalence of aspirin use and determined its risk factors among patients who presented to the ER at King Fahad Hospital in Al-Hofuf, with suspected cardiac disease. Data from this study suggest that there was a high prevalence of aspirin use among the sample population. Approximately 65% of the patients were currently using aspirin, 11% used it previously and 2% were planning to use it (overall prevalence: 78%). However, the unwarranted use of aspirin was found to be low as only 5.1% were using aspirin upon recommendation of a non-physician such as family or friends. These findings are consistent with a paper written by Liu et al. [11]. Based on their accounts, 61.7% of older American patients with diabetes mellitus (DM) were aspirin users whereas 42.2% of those without DM were also reported to be taking the medication. The prevalence of unwarranted use of aspirin in our study was relatively low compared to a study by Chen et al. [12]. According to their reports, the misuse of aspirin has been reported by 31% of patients who were at low risk of cardiovascular events while the overall prevalence of aspirin use was 38.6% which was similarly documented among Irish patients with CVD [13].

In our study, the factors that were associated with regular aspirin were advanced age and having associated chronic diseases. However, there were no differences in regular aspirin use based on gender, educational level, and level of physical activity. In the United States [4], age-adjusted aspirin use for primary prevention increased during the study period (1980-2009) ranging from 1% to 21% in men and 1% to 12% in women which was highest in patients aged 65 to 74 years, whereas for secondary prevention, age-adjusted aspirin use rose from 19% to 74% in men and 11% to 64% in women. In Denmark [14], aspirin use was independently associated with age, however, researchers pointed out that due to the widespread use of aspirin including the concomitant use of other medications specifically among older adults, the incidence of major bleeding became an issue suggesting that appropriate measures should be in place to limit the misuse of aspirin. Contradicting these reports, a study conducted among Chinese patients [11], found that among patients with diabetes, the likelihood of aspirin use did not differ significantly by age, and the level of cholesterol was also negatively associated with aspirin use while patients in low-level hospitals were more likely to exhibit inappropriate use of the medication.

Regarding the awareness of the patients toward the potential complication of inappropriate use of aspirin, we discovered that some patients believed the benefits of taking aspirin outweigh the potential risk (43%), however, they were less aware that peptic ulcers are possible complications of taking aspirin (21%) and only 7% were aware that there were drugs that should not be taken by patients who regularly take aspirin. Incidentally, we found out that these subgroups of the population were significant users of aspirin. The awareness of the appropriate use of aspirin is important to alleviate any potential complications, which underlines the importance of proper patient education by healthcare providers. It can be observed that 58% of our participants had a previous history of hospitalization due to stroke or cardiac condition with coronary artery disease as the most common cause of hospitalization (51.7%).

Ignoring the potential risks of regular aspirin use, many studies proved the effectiveness of aspirin in CVD prevention whether it is primary or secondary. For example, Guirguis-Blake et al. [6] reported that in trials with aspirin doses of 100 mg or less per day, the reduction in nonfatal myocardial infarction (MI) benefit persisted and a 14% reduction in nonfatal stroke benefit was noted and older adults assumed to achieve better MI benefits after the last follow up. Among UK patients [8], who were at CVD risk, the risk of major adverse cardiovascular events significantly decreased in groups taking aspirin compared to placebo. They further added that aspirin significantly reduced the risk of MI for a treatment duration of five years or less.

However, despite some of aspirin's great benefits, many studies pointed out the risk of inappropriate use. For instance, Christensen et al. [14] noted that there were similar incidences of major bleeding among patients who were taking aspirin for primary or secondary prevention. This has been mirrored by the study of Bowman et al. [15] showing an incidence of major bleeding occurring in 4.1% of aspirin users higher than in patients in the placebo group (3.2%). In a review written by Raber et al. [5], it was discovered that although early trials showed aspirin provided great benefits in the reduction of MI and stroke, a recent three large randomized clinical trials of aspirin for the primary prevention of CVD showed little or no benefit and have even suggested net harm for cardiovascular diseases. The author suggested re-evaluating the role of aspirin in the primary prevention of CVD by taking into consideration of the available data from previous and current clinical trials.

## Conclusions

The use of aspirin was prevalent among the sample population, but their unwarranted use was low. Older patients with chronic diseases who believed in its great benefit and tended to ignore its complications are the most common users of aspirin. The misuse of aspirin for CVD prevention is a widespread practice among the general population and should be regulated based on existing guidelines. Excessive usage of such medication may cause adverse events including gastrointestinal bleeding. More research is recommended to determine the prevalence and the factors associated with unwarranted use of aspirin in patients with CVD in our region.

## References

[1] Virani SS, Alonso A, Benjamin EJ (2020). Heart Disease and Stroke Statistics-2020 update: a report from the American Heart Association. Circulation.

[2] Reamy BV, Williams PM, Kuckel DP (2018). Prevention of cardiovascular disease. Prim Care.

[3] Patrono C, Baigent C (2019). Role of aspirin in primary prevention of cardiovascular disease. Nat Rev Cardiol.

[4] Luepker RV, Steffen LM, Duval S, Zantek ND, Zhou X, Hirsch AT (2015). Population trends in aspirin use for cardiovascular disease prevention 1980-2009: the Minnesota heart survey. J Am Heart Assoc.

[5] Raber I, McCarthy CP, Vaduganathan M (2019). The rise and fall of aspirin in the primary prevention of cardiovascular disease. Lancet.

[6] Guirguis-Blake JM, Evans CV, Senger CA, O'Connor EA, Whitlock EP (2016). Aspirin for the primary prevention of cardiovascular events: a systematic evidence review for the U.S. Preventive Services Task Force. Ann Intern Med.

[7] Richman IB, Owens DK (2017). Aspirin for primary prevention. Med Clin North Am.

[8] Kunutsor SK, Seidu S, Khunti K (2017). Aspirin for primary prevention of cardiovascular and all-cause mortality events in diabetes: updated meta-analysis of randomized controlled trials. Diabet Med.

[9] Gelbenegger G, Postula M, Pecen L (2019). Aspirin for primary prevention of cardiovascular disease: a meta-analysis with a particular focus on subgroups. BMC Med.

[10] Judge C, Ruttledge S, Murphy R (2020). Aspirin for primary prevention of stroke in individuals without cardiovascular disease-A meta-analysis. Int J Stroke.

[11] Liu EY, Al-Sofiani ME, Yeh HC, Echouffo-Tcheugui JB, Joseph JJ, Kalyani RR (2021). Use of preventive aspirin among older US adults with and without diabetes. JAMA Netw Open.

[12] Chen Y, Yin C, Li Q, Yu L, Zhu L, Hu D, Sun Y (2021). Misuse of aspirin and associated factors for the primary prevention of cardiovascular disease. Front Cardiovasc Med.

[13] Moriarty F, Barry A, Kenny RA, Fahey T (2021). Aspirin prescribing for cardiovascular disease in middle-aged and older adults in Ireland: findings from the Irish longitudinal study on ageing. Prev Med.

[14] Christensen MB, Jimenez-Solem E, Ernst MT, Schmidt M, Pottegård A, Grove EL (2021). Low-dose aspirin for primary and secondary prevention of cardiovascular events in Denmark 1998-2018. Sci Rep.

[15] Bowman L, Mafham M, Wallendszus K (2018). Effects of aspirin for primary prevention in persons with diabetes mellitus. N Engl J Med.

